# Optimizing treatment outcomes: immune tolerance induction in Pompe disease patients undergoing enzyme replacement therapy

**DOI:** 10.3389/fimmu.2024.1336599

**Published:** 2024-04-23

**Authors:** Hui-An Chen, Rai-Hseng Hsu, Ching-Ya Fang, Ankit K. Desai, Ni-Chung Lee, Wuh-Liang Hwu, Fuu-Jen Tsai, Priya S. Kishnani, Yin-Hsiu Chien

**Affiliations:** ^1^ Department of Medical Genetics, National Taiwan University Hospital, Taipei, Taiwan; ^2^ Department of Pediatrics, National Taiwan University Hospital, Taipei, Taiwan; ^3^ Department of Pediatrics, National Taiwan University College of Medicine, Taipei, Taiwan; ^4^ Division of Medical Genetics, Department of Pediatrics, Duke University School of Medicine, Durham, NC, United States; ^5^ Center for Precision Medicine, China Medical University Hospital, Taichung, Taiwan; ^6^ Department of Medical Research, China Medical University Hospital, Taichung, Taiwan

**Keywords:** Pompe disease, enzyme replacement therapy, alglucosidase alfa, immunomodulation therapy, anti-drug antibody

## Abstract

**Introduction:**

Pompe disease, a lysosomal storage disorder, is characterized by acid α-glucosidase (GAA) deficiency and categorized into two main subtypes: infantile-onset Pompe disease (IOPD) and late-onset Pompe disease (LOPD). The primary treatment, enzyme replacement therapy (ERT) with recombinant human GAA (rhGAA), faces challenges due to immunogenic responses, including the production of anti-drug antibody (ADA), which can diminish therapeutic efficacy. This study aims to assess the effectiveness of immune tolerance induction (ITI) therapy in cross-reactive immunologic material (CRIM)-positive Pompe disease patients with established high ADA levels.

**Method:**

In a single-center, open-label prospective study, we assessed ITI therapy’s efficacy in Pompe disease patients, both IOPD and LOPD, with persistently elevated ADA titers (≥1:12,800) and clinical decline. The ITI regimen comprised bortezomib, rituximab, methotrexate, and intravenous immunoglobulin. Biochemical data, biomarkers, ADA titers, immune status, and respiratory and motor function were monitored over six months before and after ITI.

**Results:**

This study enrolled eight patients (5 IOPD and 3 LOPD). After a 6-month ITI course, median ADA titers significantly decreased from 1:12,800 (range 1:12,800-1:51,200) to 1:1,600 (range 1:400-1:12,800), with sustained immune tolerance persisting up to 4.5 years in some cases. Serum CK levels were mostly stable or decreased, stable urinary glucose tetrasaccharide levels were maintained in four patients, and no notable deterioration in respiratory or ambulatory status was noted. Adverse events included two treatable infection episodes and transient symptoms like numbness and diarrhea.

**Conclusion:**

ITI therapy effectively reduces ADA levels in CRIM-positive Pompe disease patients with established high ADA titers, underscoring the importance of ADA monitoring and timely ITI initiation. The findings advocate for personalized immunogenicity risk assessments to enhance clinical outcomes. In some cases, prolonged immune suppression may be necessary, highlighting the need for further studies to optimize ITI strategies for Pompe disease treatment. ClinicalTrials.gov NCT02525172; https://clinicaltrials.gov/study/NCT02525172.

## Introduction

Pompe disease, a rare genetic disorder, results from a deficiency in the enzyme acid alpha-glucosidase (GAA), leading to glycogen accumulation in various tissues, particularly the muscles (1). The abnormal buildup of glycogen causes muscle deterioration and weakness, manifesting as cardiomyopathy, mobility impairments, and, eventually, respiratory failure. Pompe disease is divided into two main subtypes: infantile-onset Pompe disease (IOPD), characterized by the development of hypertrophic cardiomyopathy (HCM), and late-onset Pompe disease (LOPD), which does not exhibit HCM (2, 3). The primary therapeutic approach for Pompe disease is enzyme replacement therapy (ERT) with recombinant human GAA (rhGAA) (4–6).

While ERT is the standard Pompe disease treatment, its effectiveness is limited, particularly in advanced stages (7). The development of anti-rhGAA immunoglobulin G (IgG) antibodies can diminish ERT’s efficacy, resulting in suboptimal clinical outcomes (8), with antibody titers as low as 1:12,800 affecting treatment effectiveness. High-sustained rhGAA IgG antibody titers (HSAT), defined as titers of ≥ 1:12,800, further complicate the treatment landscape (8–10). A patient’s ability to produce endogenous GAA, which is defined as cross-reactive immunologic material (CRIM) status, correlates with the development of anti-drug antibodies (ADA) (11). Based on the presence of endogenous GAA, IOPD patients are classified as either CRIM-positive or CRIM-negative. Significantly, a majority of CRIM-negative and approximately one-third of CRIM-positive IOPD patients are prone to developing HSAT, which may lead to clinical deterioration (8, 10, 12).

Immune modulation has emerged as a promising strategy to mitigate the ADA response and enhance the efficacy of ERT, particularly in CRIM-negative IOPD patients (13, 14). Various preventative and therapeutic approaches have been employed in treating Pompe disease patients. For CRIM-negative IOPD patients, initiating immune tolerance induction (ITI) therapy concurrent with ERT has become the standard of care, aiming to prevent ADA formation and minimize their negative impact on treatment (13, 15). Several immunomodulatory agents, including rituximab, methotrexate, intravenous immunoglobulin (IVIg), and omalizumab, have been tested as monotherapy or combined, achieving varying degrees of success (15–18). Consequently, prophylactic immunomodulation prior to ERT initiation is commonly used in CRIM-positive IOPD patients at high risk of ADA development or those with indeterminate CRIM status requiring urgent treatment (10, 19).

However, consensus on the impact of ADA on therapeutic efficacy and the necessity of ITI for LOPD patients remains elusive. Approximately 10 percent of LOPD patients develop ADA, with some showing HSAT associated with clinical decline (20). Conversely, other studies indicate that long-term ERT in LOPD patients leads to high ADA titer, but the immune responses to rhGAA following treatment do not significantly affect its efficacy (21). The diverse characteristics and varied treatment responses among LOPD patients complicate the assessment of ERT effects and ADA’s influence on this group (22).

This study aims to offer insights into immunogenicity in CRIM-positive patients and explore the role of immunomodulation in CRIM-positive Pompe patients exhibiting high ADA levels undergoing ERT. The study encompassed both IOPD and LOPD patients who developed high ADAs and received therapeutic ITI treatment. Our therapeutic immunomodulation therapy, incorporating rituximab, methotrexate, IVIg, and bortezomib, has shown success in eliminating IgG antibodies in patients with HSAT, thereby establishing immune tolerance (14).

## Materials and methods

### Study design

We conducted a single-center, open-label, prospective study to evaluate the efficacy of therapeutic immunomodulation in patients diagnosed with Pompe disease from 2015 onwards. All participants received alglucosidase alfa. Since 2019, those prescribed a higher dose of ERT were pre-treated with prophylactic transient low-dose methotrexate (23). ADA titers were monitored quarterly, alongside clinical response and biomarkers, including creatine kinase (CK) and urinary glucose tetrasaccharide (urinary Glc_4_), evaluated every 3 to 6 months.

### Patient selection

Eligibility criteria required patients to have (1) persistently elevated ADA titers of ≥1:12,800 on two separate occasions, three months apart, and (2) an observable clinical deterioration in either motor or respiratory function based on annual interdisciplinary assessments. Decline in motor function was characterized by escalating weakness or less-than-expected gains in patients younger than 6 years old. Respiratory decline was defined by an increased reliance on respiratory support. Exclusion criteria included (1): patients at risk for hepatitis B/C reactivation (2), those at risk for latent tuberculosis infection or with a history of contact with individuals being actively treated for tuberculosis (TB) (3), patients who had received any investigational product other than alglucosidase alfa within 30 days before study enrollment (4), pregnancy or lactation, and (5) those who had received live vaccination within one month before enrollment.

### Immunomodulation

Before ITI initiation, patients underwent screening for infections such as hepatitis B, hepatitis C, and TB. Our protocol, modified from Duke University’s “Immune Response Protocol” (24), involved a six-month immunomodulation cycle with rituximab, oral methotrexate administered before ERT sessions, monthly IVIg, and four doses of bortezomib post-first ERT session. Patients with inadequate ADA reduction after six months, defined as ADA ≥ 1:6,400, underwent a second round of ITI cycle.

### Efficacy and outcome measurements

A thorough baseline clinical evaluation was conducted, capturing essential data before high ADA titers development. This included parameters such as *GAA* pathogenic variants, Pompe disease classification, CRIM status, ERT initiation age, ERT dosages, overall and ventilator-free survival, ambulatory status, feeding status, and motor status. Patients diagnosed after developing symptoms were categorized as clinical cases rather than newborn screening (NBS) cases. Among patients with IOPD, all NBS cases received ERT within 6-30 days of age, while clinical cases initiated ERT after the neonatal stage. Biochemical data, biomarkers, and other relevant information, such as lymphocyte counts, immunoglobulin status, and ADA levels, were obtained at baseline and then tracked monthly until one month after the conclusion of the study to monitor toxicity and evaluate treatment efficacy. Motor function was assessed by an experienced physical therapist using the Peabody Developmental Motor Scales-2 (PDMS-2) for patients under six years old (25).

The study’s primary endpoint was ADA titer reduction post-ITI. Clinical outcomes were also evaluated through motor function and respiratory status assessments. Data up to patients’ latest check-ups or before transitioning to other rhGAA treatments were recorded to understand the persistence of immune tolerance better.

### Safety assessment

All adverse events (AEs), serious adverse events (SAEs), and infusion-associated reactions were meticulously monitored and recorded. ITI safety was measured by infection frequency and severity of infection episodes warranting hospitalization. The dosage and regimen were adjusted according to the protocol when medication toxicity was suspected.

### Statistical analysis

ADA titer comparisons before and after treatment were analyzed using the Wilcoxon signed rank test, chosen specifically for our small patient cohort (n=8), where a normal data distribution cannot be assumed. A *p*-value of <0.05 was considered indicative of statistical significance. Statistical analyses were performed using SPSS 22 for Windows (IBM Corp., Armonk, NY, IBM Corp.).

### Ethics approval

This study secured approval from the National Taiwan University Hospital’s institutional review board (NTUH-IRB; No. 201504036MIPB). Participating patients or their legal guardians gave written informed consent before enrollment. We ensured that all participants were fully informed about the nature of the combined treatment, its experimental status, the potential risks, and benefits compared to standard ERT alone. All subjects understood that the decision to implement ITI was medically driven based on individual histories and the potential for improved quality of life.

## Results

### Patient demographics

**Figure 1:**
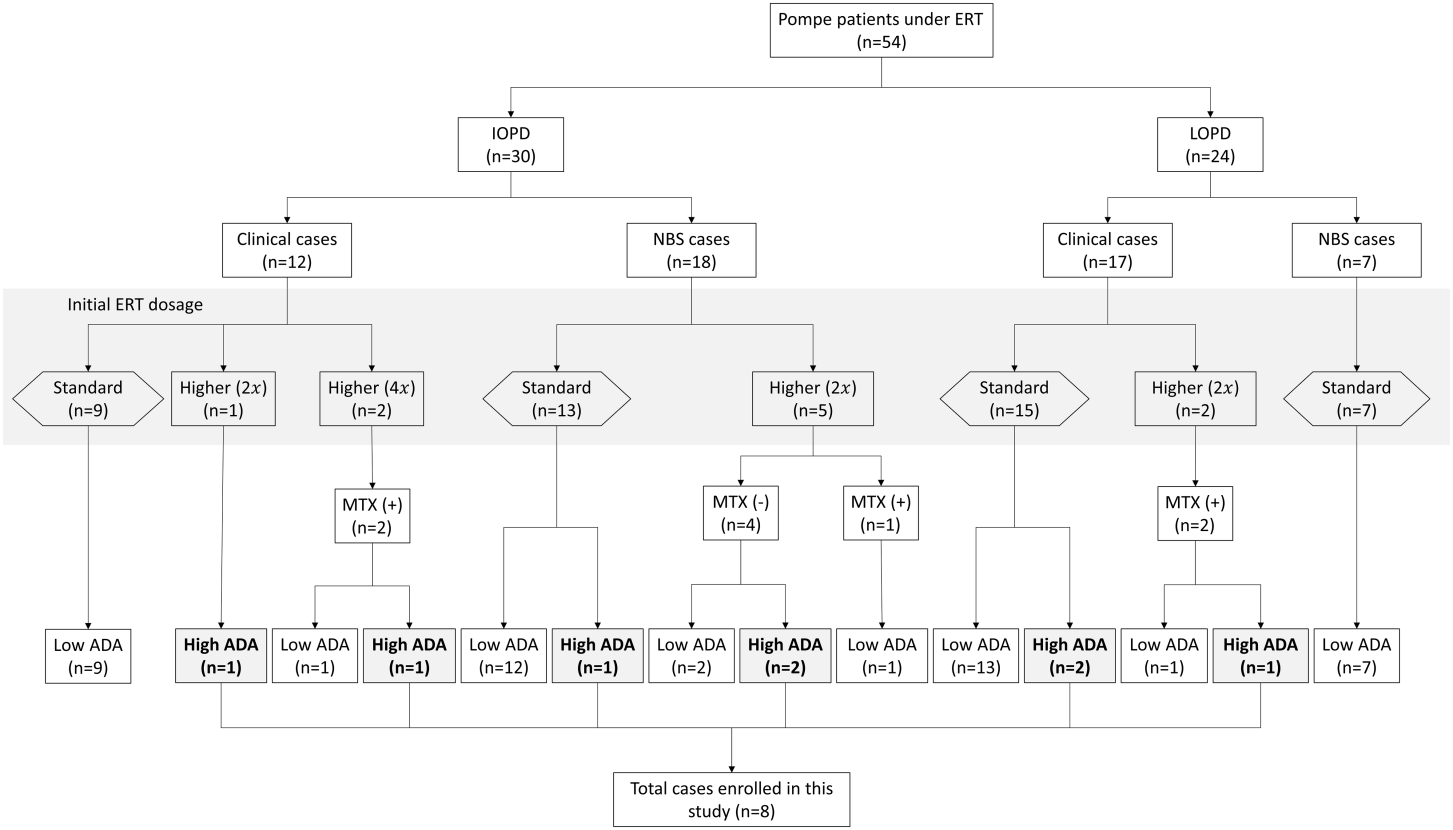
Classification and ADA development in pompe disease patients ERT. ERT, enzyme replacement therapy; IOPD, infantile-onset Pompe disease; LOPD, late-onset Pompe disease; MTX, methotrexate; ADA, anti-drug antibodies; ITI, immune tolerance induction therapy. This flowchart depicts the categorization and distribution of Pompe patients under enzyme replacement therapy (ERT). The chart distinguishes between classic infantile-onset Pompe disease (IOPD) and late-onset Pompe disease (LOPD). The patients are further categorized based on the initial ERT dosage, methotrexate (MTX) usage, and anti-drug antibody (ADA) titers. “NBS” refers to newborn screening, indicating patients diagnosed via neonatal screening protocols. “Clinical cases” show those diagnosed based on clinical symptoms. The multiplier in parentheses (e.g., 2x) signifies the fold increase in ERT dosage relative to the standard dose. A “Higher (2x)” dosage indicates that the patient received a higher dosage of 40 mg/kg every other week, while “Higher (4x)” indicates that the patient received a weekly dose of 40 mg/kg. The study enrolled eight patients who developed high ADA titers for further analysis.

From 2006 to 2023, 54 patients received ERT with alglucosidase alfa at NTUH, consisting of 30 IOPD and 24 LOPD cases (**Figure 1**). Among these, eight patients, comprising 3 LOPD (Subject A1-A3) and 5 IOPD (Subject B1-B5), represented 15% of the cohort, exhibited persistently elevated ADA titers of ≥ 1:12,800 (**Table 1**). All eight patients met the predefined criteria for clinical motor decline or suboptimal treatment response, necessitating ITI initiation, including 4 males and 4 females. The initial rhGAA dose was found to influence the development of ADA. A lower incidence of high ADA titers—3 out of 44 patients (6.8%)—was observed among those treated with the standard rhGAA dose of 20 mg/kg every other week (Q2W). In contrast, a higher incidence—5 out of 10 patients (50%)—occurred in those who received a higher initial dose of rhGAA. The higher doses included 40 mg/kg Q2W or 40 mg/kg weekly (QW), representing two or four times the standard dose. Since 2019, for patients who received these higher doses, prophylactic methotrexate was administered to mitigate ADA development. Among the eight individuals (6 IOPD, 2 LOPD) who received 40 mg/kg Q2W, a lower ADA development rate (33.3%, 1 out of 3) was noted in patients treated with prophylactic methotrexate compared to those without it (60%, 3 out of 5). ADA response rates between clinically diagnosed and NBS-diagnosed IOPD patients were similar: 2/12 (16.7%) clinically diagnosed and 3/18 (16.7%) NBS-diagnosed IOPD developed high ADA titers.

**Table 1:** Demographic and clinical data of pompe patients in this study.

Subject	Sex	Classification	Case condition	*GAA* variant		Age of ERT start (Year)	Initial ERT dose (mg/kg)	Age at high ADA* development (Year)	Duration of ERT before high ADA* (Year)	Duration of ERT before ITI (Year)	Age of ITI initiation (Year)	Clinical decline
				Allele 1	Allele 2							
A1	M	LOPD	Clinical	c.[2228A>C;1726G>A]	c.[2228A>C;1726G>A]	22	20 Q2W	23.7	1.7	3.3	25.3	Gross motor function decline
A2	M	LOPD	Clinical	c.[1935C>A;1726G>A]	c.1781G>A	3.2	20 Q2W	9.8	6.6	7.1	10.2	Gross motor function decline
A3	M	LOPD	Clinical	c.[1935C>A;1726G>A]	c.[842G>T;1726G>A]	7	40 QW	7.3	0.3	0.7	7.7	Suboptimal gross improvement
B1	F	IOPD	NBS	c.[1935C>A;1726G>A]	c.[1411_1414del;752C>T;761C>T]	0.1	20 Q2W	7.9	7.8	9.2	9.4	Gross motor function decline
B2	F	IOPD	NBS	c.[1935C>A;1726G>A]	c.2024_2026del	0.02	40 Q2W	1.4	1.4	1.6	1.7	Suboptimal gross development
B3	F	IOPD	Clinical	c.[1935C>A;1726G>A]	c.1340_1350del	0.3	40 Q2W	0.5	0.2	0.8	1.1	Increased respiration support level
B4	M	IOPD	NBS	c.[1935C>A;1726G>A]	c.[1935C>A;1726G>A]	0.1	40 Q2W	1.6	1.5	2.2	2.3	Decrease in gross motor percentile
B5	F	IOPD	Clinical	c.[1935C>A;1726G>A]	c.[1001G>A;1726G>A]	0.3	40 QW	0.4	0.1	0.6	0.9	Suboptimal gross development

All patients were CRIM+.

*High ADA is defined as ADA titers ≥12,800.

M, male; F, female; IOPD, classic infantile onset Pompe disease; LOPD, late-onset Pompe disease; NBS, newborn screening; y, year; ERT, enzyme replacement therapy; ADA, anti-drug antibody; Q2W, every other week; ITI, immune induction therapy.

Three patients were diagnosed with LOPD (Subject A1-A3) with muscle weakness noted during early adolescence, early infancy, and four years of age, respectively. None exhibited any cardiac manifestations. The remaining five were diagnosed with classic IOPD between 8 days and three months of age, primarily presenting with significant cardiac complications. Apart from Subject A1, all carried at least one allele with the c.1935C>A (p.D645E) variant in the *GAA* gene. All IOPD patients were confirmed CRIM-positive through western blotting, described previously, or genotyping prediction (26). A higher prevalence of high-dose ERT (40 mg/kg Q2W or QW) initiation (80% in IOPD and 33% in LOPD patients) was observed. The median ages upon enrollment were 1.7 years for IOPD patients and 10.2 years for LOPD patients. Subjects initially on the standard ERT dose (A1, A2, and B1) developed high ADAs after 1.7, 6.6, and 7.8 years of treatment, respectively, while those on higher doses exhibited a median duration to high ADAs of 0.3 years (range 0.1-1.4 years). Two (Subject A3 and B5) of the five subjects on higher initial doses received prophylactic methotrexate before their first ERT infusion. Notably, despite initiating ERT at eight days of age with a dosage of 40 mg/kg Q2W, Subject B2 exhibited a significant decline in motor function based on PDMS-2 evaluation before study enrollment.

The baseline condition of the eight patients, defined as the state before developing high ADA, is detailed in **Table 2**. None of the patients experienced normalization of biomarkers under their original rhGAA ERT regimens. Specifically, Subject A2’s ADA titers reached 1: 102,400, and Subject A3 had the highest peak ADA level at 1:204,800; both decreased to 1:51,200 before starting ITI. Subject B3 showed a peak ADA level of 1:51,200, which fell to 1:12,800 before starting ITI. Despite a potential decrease in ADA levels before ITI enrollment, ADA titers remained significantly elevated, warranting the initiation of ITI. The remaining patients had persistently high ADA titers exceeding 1:12,800 for more than six months. Prior to ITI, all patients showed elevated levels of CK with a median of 708 U/L (range 517-2500 U/L), urinary Glc_4_ with a median of 32.46 mmol/mol CRE (range 17.86-63.66 mmol/mol CRE), and ADA titer levels with a median of 1:12,800 (range 1:12,800-1:51,200). Subjects B1, who was initially ambulatory independently, required assistance for ambulation at the time of ITI initiation. Additionally, Subjects B1 and B3 exhibited an increased need for bi-level positive airway pressure (BiPAP) support compared to their conditions at baseline.

**Table 2:** Immunomodulation therapy impact on ADA titers and clinical status.

Subject (course)	ADA titers			ALG dose (mg/kg)			CK (U/L)			Glc_4_			Ambulation status			Respiration status		
	Baseline	Pre-ITI	End of ITI	Baseline	Pre-ITI	End of ITI	Baseline	Pre-ITI	End of ITI	Baseline	Pre-ITI	End of ITI	Baseline	Pre-ITI	End of ITI	Baseline	Pre-ITI	End of ITI
A1 (1)	1:6400	1:12800	1:6400	20 Q2W	20 Q2W	20 Q2W	*NA*	708	635	*NA*	63.66	30.99	Independently ambulatory	No change	No change	Room air	No change	No change
A1 (2)	*NAp*	1:6400	1:3200	*NAp*	20 Q2W	20 Q2W	*NAp*	635	486	*NAp*	30.99	32.69	*NAp*	No change	No change	*NAp*	Night BiPAP	No change
A2	1:6400	1:51200	1:12800	20 Q2W	20 QW	20 QW	851	613	1480	42.63	26.66	20.81	Independently ambulatory	No change	No change	Room air	No change	No change
A3	Negative	1:51200	1:1600	40 QW	20 Q2W	40 Q2W	437	1555	1054	48.55	50.73	70.65	Independently ambulatory	No change	No change	Night BiPAP	No change	No change
B1	1:6400	1:25600	1:3200	20 Q2W	20 Q2W	20 Q2W	2560	2500	1462	75.09	44.91	133.88	Independently ambulatory	Ambulatory with assistance	No change	Room air	Night BiPAP	No change
B2	1:6400	1:12800	1:400	40 Q2W	50 Q2W	40 QW	2217	1945	1862	*NA*	32.46	39.6	Independently ambulatory	No change	No change	Room air	No change	No change
B3	1:6400	1:12800	1:800	40 Q2W	20 QW	40 Q2W	841	907	618	25.28	33.16	68.91	*NAp*	Wheelchair-bound	No change	Night BiPAP	Full day BiPAP	No change
B4	1:6400	1:12800	1:1600	40 Q2W	40 Q2W	40 Q2W	434	628	1118	12.01	17.86	24.66	Independently ambulatory	No change	No change	Room air	No change	No change
B5	Negative	1:51200	1:1600	40 QW	40 QW	NeoGAA 40 Q2W	979	517	626	43.13	23.87	14.04	*NAp*	Ambulatory with assistance	Independently ambulatory	Room air	No change	No change

AGL, Alglucosidase alfa; NeoGAA, Avalglucosidase alfa; CK, creatine kinase; ERT, enzyme replacement therapy; Glc_4_, glucose tetrasaccharide in urine, N<4 mmol/mol CRE; QW, every week; Q2W, every other week; ADA: anti-drug antibody; NA, not available; NAp, not applicable.

*Baseline measurements were taken at the last assessment before developing high sustained or persisted ADA titer.

### Efficacy of immune modulation

**Figure 2:**
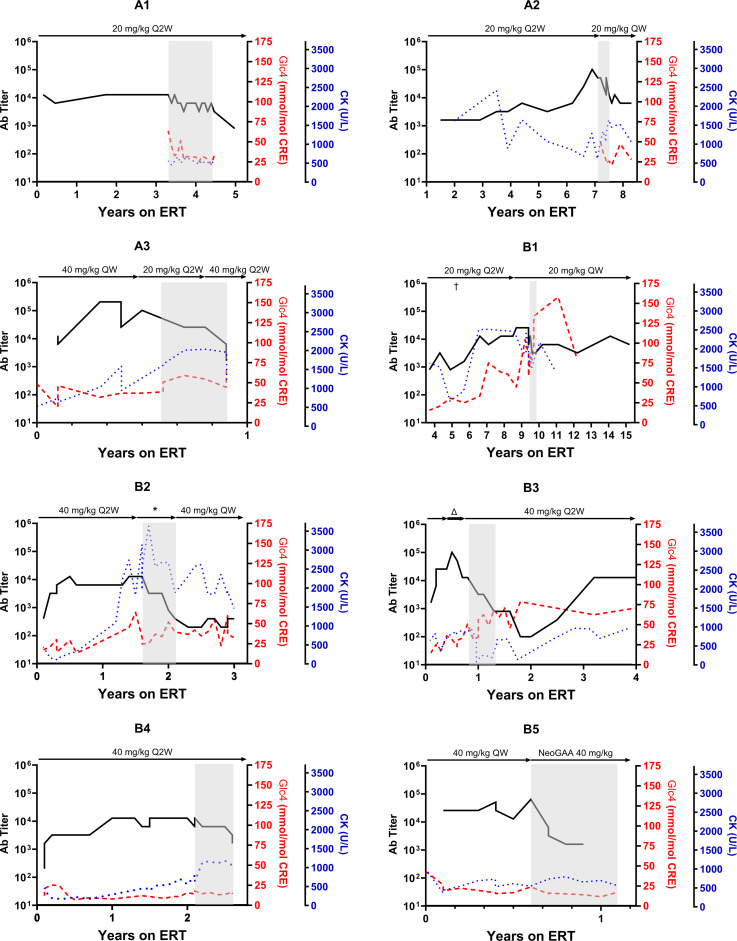
Trajectory of anti-drug antibody following alglucosidase alfa treatment in pompe disease patients. This figure illustrates the progression of anti-drug antibodies (ADA) titers over time for each patient undergoing enzyme replacement therapy (ERT), with ERT dosages indicated above each graph. The grey area in each panel indicates the period during which immune tolerance induction (ITI) therapy was conducted. For Patient B1, the ERT dosage was increased from the conventional 20 mg/kg every other week (Q2W) to a weekly 20 mg/kg (QW) for five months in the fifth year of ERT, denoted by “†.” Patient B2 received several ERT dosage modifications during the study, denoted by*, with specific dosages detailed in **Figure 3**. Patient B3 was initially administered a doubled dose of alglucosidase alfa at 40 mg/kg Q2W, which was later increased to a quadrupled dosage (40 mg/kg QW, indicated by Δ) before returning to the standard dosage after four months.

A marked reduction in ADA titers was observed at the conclusion of the planned 6-month immunomodulation therapy (**Table 2**, **Figure 2**). The median titer decreased from 1:12,800 (1:12,800 to 1:51,200) before ITI to 1:1,600 post-ITI (range: 1:400 to 1:12,800; *p-value*: 0.018). Despite experiencing the highest post-ITI ADA titer, Subject A2 improved from 1:51,200 to 1:12,800 and transitioned to the new form of rhGAA without undergoing another round of ITI. Subsequently, the ADA titer stabilized at 1:6,400 over the following nine months. Subsequently, the ADA titer stabilized at 1:6,400 over the following nine months. The rest of the participants either stabilized or experienced decreases in serum CK levels following the ITI protocol.

While four subjects maintained stable post-ITI urinary Glc_4_ levels compared to pre-ITI levels, four others (Subject A3, B1, B3, B4) showed increases from baseline and pre-ITI levels. All participants maintained their respiratory and mobility statuses by the study’s end. Notably, Subject A1, who had initially declined the recommendation of nighttime ventilation support for personal reasons, consented to nocturnal invasive ventilation during the second ITI course due to the lack of anticipated clinical improvement.

The long-term effects of the ITI are briefly listed in **Table 3**. Following the ITI period, three patients (Subject A1, B1, B3) continued with alglucosidase alfa for over a year, maintaining reduced ADA titers for 2 to 4.5 years post-ITI (**Table 3**, **Figure 2**). Subject B1 passed away at 16, six years after ITI, due to a sudden collapse at home—potentially from a seizure, with a cardiac event also considered a possible cause. The remaining patients transitioned to new forms of rhGAA within a year. Subsequently, when avalglucosidase alfa was available locally, all were switched to this newer ERT formulation (**Table 3**). Subject A2, B3, and B4 experienced recurrent high ADA levels (1:12,800) against avalglucosidase alfa after switching.

**Table 3:** The most recent condition in patients having ITI in this study.

Subject	Current age (y)	Years Post-ITI treated with original rhGAA	High ADA titers recurrence (years post ITI if yes)	Current ERT
A1	31.2	5.0	N	Avalglucosidase alfa 20 mg/kg Q2W
A2	15.8	1.0	N	Avalglucosidase alfa 40 mg/kg Q2W
A3	8.1	0	N	Avalglucosidase alfa 40 mg/kg Q2W
B1	16.0*	6.3	Y (4.5)	Alglucosidase alfa 20 mg/kg Q2W
B2	6.2	0.7	N	Avalglucosidase alfa 40 mg/kg Q2W
B3	4.8	2.8	Y (2)	Avalglucosidase alfa 40 mg/kg Q2W
B4	5.4	0	N	Avalglucosidase alfa 40 mg/kg Q2W
B5	1.5	0	N	Avalglucosidase alfa 40 mg/kg Q2W

ERT, enzyme replacement therapy; QW, every week; Q2W, every other week; ADA, anti-drug antibody. *B1 expired at the age of 16 years old. N, no recurrence; Y, with recurrence.

### Dosage adjustments and their impacts on ERT

**Figure 3:**
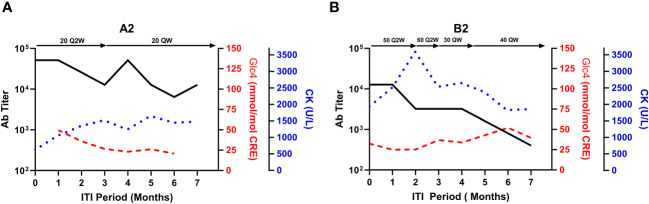
Biomarker fluctuations in relation to ERT dosage adjustments during ITI for subjects A2 and B2. This figure presents the relationship between serum CK, urinary glucose tetrasaccharide (Glc_4_), and anti-rhGAA antibody (ADA) titers against the backdrop of enzyme replacement therapy (ERT) dosage adjustments during the immune tolerance induction (ITI) period for subjects A2 **(A)** and B2 **(B)**. Notable changes in ERT dosages are indicated above each graph, with the timeline post-ITI initiation plotted on the X-axis.

During ITI, clinical deterioration indicative of inadequate ERT response prompted dose adjustments in two subjects (Subject A2 and B2). Subject A2’s dosage was increased from 20 mg/kg Q2W to 20 mg/kg QW in the second month. By the fourth month, a peak ADA titer of 1:51,200 was observed, subsequently decreasing to 1:6,400 (**Figure 3A**). Despite this, biomarker trajectories diverged; urinary Glc_4_ levels declined from 26.66 to 20.81 mmol/mol CRE, while CK levels rose from 613 to 1,480 U/L. Subject B2 experienced three dose adjustments, resulting in a steady ADA titer decrease (**Figure 3B**). Urinary Glc_4_ and CK levels remained stable throughout ITI, highlighting the complexity of attributing outcomes directly to ITI or dosage changes.

### Safety evaluation

No significant deviations in liver enzymes (AST and ALT) were detected from baseline. Leukopenia was absent, and IgG levels were stable. CD19 levels became near-undetectable one month after initiating ITI, indicating effective B-cell depletion.

Three SAEs were documented, all either directly related or potentially related to ITI: one allergic reaction to rituximab and two distinct infection episodes (**Table 4**). Subject B2 developed symptoms of fever, cough, and dyspnea during the initial rituximab infusion, which were managed with antihistamines and steroids and adjusted the infusion rate. Following treatment, the patient could resume and complete the treatment the following day. This patient did not report any similar reactions in the remaining ITI courses. Subject B1 was hospitalized twice for infections, once for acute otitis media, leading to a 5-day stay, and another for influenza-related pneumonia with respiratory distress, necessitating a 7-day stay. The patient made a full recovery after both episodes. None of the participants withdrew from the study.

**Table 4:** Adverse events observed during the six-month ITI treatment.

Type of events	Patients (n=8)	No of events (n=16)
Serious AEs (n=3)		
Dyspnea	1 (12.5%)	1 (6.3%)
Pneumonia	1 (12.5%)	1 (6.3%)
Acute otitis media	1 (12.5%)	1 (6.3%)
Other TEAEs (n=13)		
Fever	3 (37.5%)	5 (31.3%)
Viral infection	1 (12.5%)	1 (6.3%)
Chills	1 (12.5%)	1 (6.3%)
Skin rash	2 (25.0%)	2 (12.5%)
Diarrhea	1 (12.5%)	2 (12.5%)
Numbness	1 (12.5%)	1 (6.3%)
Tachycardia	1 (12.5%)	1 (6.3%)

No, number, AEs, adverse events, TEAEs, treatment emergent adverse events.

Other reported AEs include fever in three (37.5%) patients, chills (12.5%), skin rash (25.0%), diarrhea (12.5%), numbness (12.5%), and tachycardia (12.5%). Specifically, subject A1 reported one episode of diarrhea and two instances of numbness, likely related to bortezomib-induced peripheral neuropathy. All AEs were transient, with patients recovering without enduring complications. During the second course of ITI, Subject A1 tested positive for latent TB (QuantiFeron test) but showed no signs of active infection, undergoing standard TB treatment with isoniazid for 270 days.

## Discussion

### ADA management and clinical outcomes

In this study, we detailed the ADA status within a CRIM-positive cohort and elucidated the outcomes following immunomodulation. Notably, high sustained antibodies, which compromise the clinical efficacy of ERT, can indeed develop in CRIM-positive patients receiving rhGAA. The critical roles of both prophylactic and therapeutic immunomodulation have been highlighted, potentially amplifying the benefits of the treatment.

Our findings underscore a marked reduction in ADA titers post-ITI among CRIM-positive Pompe patients, employing a protocol previously evidenced to bolster overall survival and enhance respiratory functions in both CRIM-negative IOPD patients and CRIM-positive patients with HSAT (14, 15). Nonetheless, while ADA levels demonstrated substantial improvement, key biomarkers such as serum CK levels and urinary Glc_4_ did not consistently reflect this decline, nor was there a discernable improvement in either motor or respiratory function. This observation aligns with Owens et al., who also found no significant clinical benefit of ITI therapy with bortezomib, methotrexate, and IVIg in two IOPD patients with high and persistent ADA levels (16). The subpar clinical outcomes observed, even in the face of reduced ADA levels, suggest that other determinants, including the disease’s severity, the timing of therapeutic commencement, and rhGAA dosing, might exert a more pronounced effect on treatment results. For example, in our cohort, Subject B3 already used a wheelchair and required full-day BiPAP support before initiating ITI, while Subject B1 ambulated with assistance and required night-time BiPAP. While we do not see substantial improvements using current evaluation methods, this may be attributed to the absence of sensitive evaluation tools in advanced cases rather than the inadequacy of these treatment methods. Early treatment and the timely initiation of ITI remain crucial for improving the efficacy of ERT in Pompe patients. Regular monitoring of ADA titers is pivotal for the early identification of patients with persistently elevated ADA levels, enabling proactive strategies to mitigate the impact of harmful IgG antibodies on ERT efficacy.

### Early intervention and dosage considerations: preventing ADA development

Early intervention measures, such as prophylactic ITI before ERT, can potentially prevent antibody development in patients at risk of high ADA titers post-ERT (13, 23). Identifying and initiating ITI in treatment-naïve patients at risk is beneficial, rather than addressing entrenched cases where patients have already developed high ADA titers. However, discerning high-risk individuals, especially among CRIM-positive patients, posed a significant challenge within our cohort. Intriguingly, our data revealed an elevated occurrence of high ADA titers among patients who initiated ERT at a higher dosage (50%) compared to those receiving a standard dosage (6.8%), irrespective of ERT duration. Furthermore, prophylactic methotrexate appeared to reduce the occurrence of high ADA titers. However, this finding was based on our single center experience and necessitates further scrutiny through comprehensive research. Given the potential for enhanced benefits of prophylactic ITI in CRIM-positive patients, particularly those receiving higher initial doses of ERT. It is crucial to develop personalized risk evaluations that effectively predict immunogenicity.

### Personalization of therapeutic ITI: regimen protocols and duration

ITI protocols for Pompe disease have been a topic of significant discussion; however, there has yet to be a consensus on personalized risk assessment and treatment strategies, particularly regarding drug selection. For example, Messinger et al. reported the successful total eradication of IgG antibodies in two CRIM-negative, non-HSAT patients using a combination regimen with rituximab, methotrexate, and IVIg (15, 27), while Poelman et al. reported the efficacy of ITI using bortezomib, rituximab, rapamycin, and IVIg in one CRIM-positive and two CRIM-negative IOPD patients (13). Using weekly IVIg doses at 1 g/kg as a single agent for 20 weeks has also been effective (18). Our study contributes to the evidence by showcasing the effectiveness of a combined regimen of bortezomib, rituximab, methotrexate, and IVIg over six months, which sustained effects for up to 4.5 years. Although we also identified potential risks, including infection episodes and suspected bortezomib-induced side effects, those effects were transient and managed with pre-medication.

Our study, with target ADA titers below 1:6,400 after a six-month ITI course, highlights the significance of ADA response monitoring post-ITI and the potential need for prolonged ITI durations or intensified treatment regimens. Subject A2 experienced a reduction in ADA titers to 1:12,800 following ITI therapy. Despite protocol guidelines for a second ITI round, the patient transitioned to a new form of rhGAA without further immunomodulation. However, subsequent elevation in ADA titers after treatment with avalglucosidase alfa suggests the necessity for additional ITI. It is important to note that these patients may not be fully immune tolerant, having only experienced a transient decrease in ADA titers; ongoing monitoring is critical. An alternative approach might be starting with two cycles of bortezomib of the ITI protocol. However, a threshold of ADA titers requiring a bortezomib rescue dose has yet to be established. Subjects B3 and B4, despite reaching anticipated ADA titer targets, experienced a resurgence to high levels (1:12,800) after receiving avalglucosidase alfa, suggesting that continuous immunotherapy after the 6-months course as protocol may be beneficial in such cases. Further research is crucial to identify optimal ITI strategies through personalized assessments and to determine the most suitable initiation times for therapy, with adjustments based on individual ADA responses critical for enhancing ITI therapeutic outcomes.

### Therapeutic ITI in LOPD: debates and outcomes

The efficacy of therapeutic ITI in LOPD remains debated due to the uncertain impact of ADA on treatment outcomes (28, 29). However, recent studies have shown promising results of therapeutic ITI in LOPD patients with HSAT (30, 31). In our study, three LOPD patients exhibited diverse responses to ITI. Subject A1’s ADA titers decreased from 1:12,800 to 1:3,200 following a second course of ITI. While CK levels remained relatively stable, there was a noticeable improvement in urinary Glc_4_ levels, a glycogen degradation product that is a comprehensive indicator of disease burden (32). This aligns with previous research suggesting that ADA concentrations at these levels might not significantly hinder the efficacy of rhGAA (29). However, the complexity of factors influencing patient outcomes, including dosage variations and the extent of preexisting muscle damage, emphasizes the need for customized ITI protocols for individual patient profiles.

### Limitations

Our prospective therapeutic study on ITI therapy provides valuable insights; however, it is important to consider the limitations and their potential impact on our findings. Firstly, the delayed initiation of the ITI protocol meant some patients had already experienced prolonged periods of elevated ADA levels before ITI, which could have led to an underestimation of its efficacy. For the younger subjects (Subject B2-B5), ITI was initiated swiftly upon the detection of elevated ADA titers, thereby minimizing the duration of high ADA exposure and potentially mitigating its impact. Recent reports have also addressed the importance of regular monitoring of ADA titers across both IOPD and LOPD patients (24, 30). Secondly, our study’s reliance on conventional clinical outcome measures, such as the 6MWT and FVC, may not be sensitive enough to discern subtle improvements within the six-month study duration. Although some patients likely benefitted clinically from ITI, adopting more sensitive evaluative methods is essential to capture changes in short-period studies accurately. Another challenge is our study’s relatively small sample size, which limits the broader applicability of our findings. Practices and patient demographics can vary significantly across centers, and our findings may not be directly applicable to broader populations due to these variations.

## Conclusions

In conclusion, our research indicates that ITI effectively reduces ADA titers in both IOPD and LOPD patients. We observed improvements in urinary Glc_4_ levels, yet the direct clinical benefits of ITI, particularly in enhancing muscle and respiratory functions, remain uncertain. This ambiguity could be attributed to several factors, including the inability of ITI to reverse pre-existing muscle damage, the limited sensitivity of our evaluation tools, and the necessity for extended follow-up periods. Our study underscores the critical importance of continuous ADA titers monitoring in CRIM-positive IOPD and LOPD patients undergoing ERT. Additionally, the duration of HSAT before initiating ITI may also influence treatment efficacy. Given the disease’s heterogeneity and the constrained sample size of our study, there’s a pressing need for a unified strategy to identify Pompe disease patients at an increased risk of ADA development, and to initiate ITI at the earliest time.

Further research with larger, multicenter, and more homogeneous cohorts is essential to validate our findings and assess the efficacy of ITI therapy across diverse patient populations. Such research endeavors will facilitate the development of more efficient, tailored treatment regimens for this complex condition.

## Data availability statement

The raw data supporting the conclusions of this article will be made available by the authors, without undue reservation.

## Ethics statement

The studies involving humans were approved by National Taiwan University Hospital’s Institutional Review Board. The studies were conducted in accordance with the local legislation and institutional requirements. Written informed consent for participation in this study was provided by the participants’ legal guardians/next of kin.

## Author contributions

HC: Data curation, Formal analysis, Investigation, Visualization, Writing – original draft. RH: Data curation, Writing – review & editing. CF: Data curation, Writing – review & editing. AD: Methodology, Resources, Writing – review & editing. NL: Data curation, Writing – review & editing. WH: Conceptualization, Resources, Supervision, Writing – review & editing. FT: Data curation, Writing – review & editing. PK: Conceptualization, Methodology, Resources, Writing – review & editing. YC: Conceptualization, Data curation, Funding acquisition, Methodology, Project administration, Resources, Supervision, Writing – original draft.
